# Une métastase cérébrale révélée par une otomastoïdite chronique

**DOI:** 10.11604/pamj.2014.18.19.4002

**Published:** 2014-05-06

**Authors:** Brahim Eljebbouri, Ali Akhaddar

**Affiliations:** 1 Service de Neurochirurgie, Hôpital Militaire d'Instruction Mohamed V Rabat, Maroc; 2 Service de Neurochirurgie, Hôpital Militaire Avicenne, Marrakech, Maroc

**Keywords:** Otomastoïdite, métastase, cérébrale, Otomastoiditis, metastasis, Brain

## Image en medicine

Il s'agit d'un patient de 48 ans suivi depuis 4 ans au service d'ORL pour une otite séromuqueuese droite pour laquelle il a était mis plusieurs fois sous traitement médical à base de corticoïdes et d'antibiotiques locaux et systémiques. A la lumière d'un bilan radiologique ce patient a été déclaré porteur d'une métastase temporale d'un cancer primitif pulmonaire- comprimant la trompe d'eustache, lysant l'os temporal et responsable d'une fuite du liquide cérébrospinal dans le conduit auditif externe (prise à tord comme étant une otomastoïdite). Ce diagnostique de métastase a été confirmé par une biopsie stéréotaxique. Le patient est décédé 4 mois après sa première séance de radiothérapie conventionnelle. Le cancer du poumon est le premier cancer prometteur de métastases cérébrales, la localisation temporale de ces métastases est pourvoyeuse de crises comitiales avec ou sans déficit neurologique. A notre connaissance, il n'a pas été déjà décrit qu'une otomastoïdite peut révélée ce type de lésion qui reste de mauvais pronostique.

**Figure 1:**
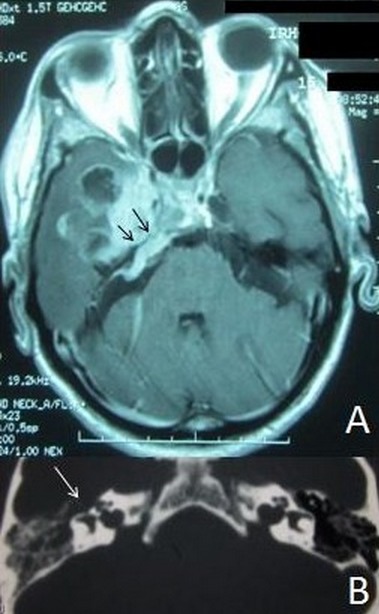
A: coupe axiale d'une IRM cérébrale en séquence pondérée T2 montrant l'obstruction de la trompe d'Eustache par le processus temporal (flêches noires); B: coupe axiale d'un scanner cérébral en fenêtre osseuse montrant la lyse osseuse du rocher droit (flêche blanche)

